# Population-level effect of potential HSV2 prophylactic vaccines on HIV incidence in sub-Saharan Africa

**DOI:** 10.1016/j.vaccine.2008.11.074

**Published:** 2009-02-05

**Authors:** Esther E. Freeman, Richard G. White, Roel Bakker, Kate K. Orroth, Helen A. Weiss, Anne Buvé, Richard J. Hayes, Judith R. Glynn

**Affiliations:** a Infectious Disease Epidemiology Unit, Department of Epidemiology and Population Health, London School of Hygiene and Tropical Medicine, Keppel Street, London, WC1E 7HT, UK; b Erasmus MC, University Medical Center Rotterdam, Rotterdam, Netherlands; c Institute of Tropical Medicine, Antwerp, Belgium

**Keywords:** HIV, HSV2, Primary prevention

## Abstract

Herpes simplex virus type-2 (HSV2) infection increases HIV transmission. We explore the impact of a potential prophylactic HSV2 vaccination on HIV incidence in Africa using *STDSIM* an individual-based model. A campaign that achieved 70% coverage over 5 years with a vaccine that reduced susceptibility to HSV2 acquisition and HSV2 reactivation by 75% for 10 years, reduced HIV incidence by 30–40% after 20 years (range 4–66%). Over 20 years, in most scenarios fewer than 100 vaccinations were required to avert one HIV infection. HSV2 vaccines could have a substantial impact on HIV incidence. Intensified efforts are needed to develop an effective HSV2 vaccine.

## 1 Introduction

HIV and Herpes simplex virus type-2 (HSV2) have a synergistic relationship. HIV affects HSV2 shedding, and the frequency and duration of clinical reactivations [1–4]. HSV2, in turn, has a strong impact on HIV transmission and acquisition, and may also affect the natural history of HIV infection [3,5–10]. A meta-analysis of longitudinal studies found HSV2 seropositivity to be associated with a risk ratio of HIV acquisition of 2.7 (95% confidence interval (CI) = 1.9–3.9) in men and 3.1 (95% CI = 1.7–5.6) in women [11] and most cross-sectional studies have found a correlation between HSV2 and frequency and/or quantity of HIV mucosal shedding [12–14].

HSV2 prevalence and the estimated proportion of HIV infections attributable to HSV2 (population attributable fraction, PAF) are very high in sub-Saharan Africa (Africa) and therefore HSV2 control could have a substantial population-level impact on the HIV epidemic [3,11]. However, two recent randomised-controlled trials of herpes suppressive therapy have failed to show an impact on HIV acquisition, perhaps because of insufficient herpes suppression due to drug dosage or inadequate adherence, or the mechanism of action of acyclovir does not reduce the role of HSV2 in HIV transmission [15,16]. HSV2 vaccines may provide a more effective means of HIV control.

An ideal prophylactic vaccine would prevent infection, although partially effective prophylactic vaccines may still be useful if they shift the threshold of infection, or if they prevent or ameliorate disease [17,18]. These vaccines could reduce HSV2 incidence by preventing infection or by reducing the infectivity of an HSV2-infected individual through reductions in shedding or clinical recurrences. Conversely, vaccines could increase HSV2 incidence if they reduced symptomatic signs of disease but had no effect on viral shedding [17,18].

Prophylactic vaccines to prevent HSV2 acquisition have been tested with limited success. The Chiron-gD2gB2-MF59 vaccine provided only temporary protection lasting a few months [18]. The GlaxoSmithKline (GSK)-gD2-alum-MPL prophylactic vaccine had no effect in men or HSV1 positive women, but in HSV1 seronegative women the risk of HSV2 infection and disease was reduced [19]. A further trial of the GSK vaccine in HSV1 negative women is ongoing. However this vaccine may not be useful in Africa because of the very high HSV1 prevalence [20].

Estimating the population-level impact of an HSV2 vaccine on HIV is complex because of its potential effects on both acquisition and transmission of HIV as well as the bi-directional relationship between HIV and HSV2. Mathematical modelling can be used to make these estimates. Few modelling studies have examined the relationship between HSV2 and HIV [21–25]. One study has explored the potential impact of HSV2 therapy on HIV incidence [26], but none have examined the impact of an HSV2 prophylactic vaccine on HIV incidence in Africa, where interventions are most needed.

The aim of this paper is to explore the potential population-level impact of prophylactic HSV2 vaccines on HIV incidence in one low and one high HIV prevalence city in sub-Saharan Africa using the *STDSIM* mathematical model.

## 2 Methods

### 2.1 Data, model and baseline model scenarios

*STDSIM* has been fitted to population-based data collected from four cities in Africa in 1997 and to available data on trends over time, as published [23,27].

*STDSIM* is an individual-based stochastic model that simulates the natural history of, and interactions between, HIV, HSV2, syphilis, gonorrhoea, chlamydia and chancroid. It has been described in detail [28,29]. *STDSIM* is able to simulate realistic sexual networks and heterogeneity between individuals in sexual behaviour and in the natural history of infection. *STDSIM* has previously been used to explore the impact of STI treatment and male circumcision for HIV-1 prevention and the heterogeneous spread of HIV-1 in Africa [23,27,30–32].

The model representation of the natural history of HSV2, and the interaction between HIV and HSV2, was parameterised based on the literature where possible, and poorly known parameter values have been subjected to sensitivity analysis [23,27]. For simplicity, HSV2 natural history was categorised into four stages: ‘primary genital herpes’, ‘early’, ‘middle’, and ‘late’. After ‘primary genital herpes’, HSV2 reactivations are modelled to occur in the ‘early’ and ‘middle’ stages with decreasing frequency with time since infection [33,34]. Clinical reactivation rates were assumed to vary widely between individuals [35]. Per-contact male-to-female (MtoF_tp_) transmission probabilities for both HIV and HSV2 were assumed to be twice those for female-to-male (FtoM_tp_) [36]. Per-contact HSV2 transmission probabilities were assumed to be highest during ‘primary genital herpes’ (MtoF_tp_ = 0.29), and lower during periods of clinical reactivation (MtoF_tp_ = 0.20) [37], although one recent study showed self-reported recurrence rates were not associated with HSV2 transmission [38]. Between clinical reactivations, a low HSV2 transmission probability was assumed during the ‘early’ (MtoF_tp_ = 0.01) and ‘middle’ (MtoF_tp_ = 0.005) stages to account for transmissions that occur during sub-clinical shedding [22,25,35,39]. We assumed no HSV2 transmission or clinical reactivations during the ‘late’ stage.

HSV2 clinical reactivation was assumed to increase HIV acquisition and transmission. The magnitude of the per-contact HSV2 cofactor effects on HIV acquisition and transmission were chosen to be at the lower end of the estimated range for genital ulcers (3–300) to account for possible residual confounding due to common risk behaviours that may inflate estimates [40] and have been subject to sensitivity analyses [23]. The cofactor effect was assumed to be the same for HIV acquisition and transmission and was 25 during primary genital herpes and 10 during recurrent HSV2 clinical reactivations. Although biologically plausible [41], no increase in the risk of HIV acquisition or transmission was assumed for the periods of sub-clinical shedding between clinical reactivations, due to the lack of definitive data. This and other poorly known assumptions were subjected to sensitivity analysis. We have previously shown that the magnitude of these per-contact cofactor effects give incidence rate ratios for the association between HIV and HSV2 prevalence, and PAFs of HIV incidence associated with HSV2, in line with empirical data [11,23,42,43]. The simulated age-adjusted relative risks of HSV2 infection for HIV incidence in Cotonou were 5.8 in males (data = 5.4, 2.2–13) and 6.6 in females (data = 7.0, 2.9–17), and in Kisumu 3.5 in males (data = 9.0, 5.1–16) and 4.4 in females (data = 4.7, 3.0–7.5) [42,43]. The simulated PAFs [31] for adults over 2 years were around 45% in Cotonou and 35% in Kisumu [23], within the range estimated in a meta-analysis that showed that 38–60% of new HIV infections in African women and 8–49% of new HIV infections in men could be due to HSV2 [11], depending on HSV2 prevalence levels that ranged from 29% to 71% in women and 5–53% in men [20]. Simulated PAFs were higher in Cotonou than Kisumu primarily because of lower rates of other cofactor STIs in Cotonou [23].

Other model parameter values, for the demography, sexual behaviour and natural history of the other STIs and their interaction with HIV were as reported in our previous publication [27], except that the mean survival time from HIV infection to death was increased from 10 to 11 years in line with the findings of a recent meta-analysis [44], and an additional increase in condom use rates was simulated in 2000 (to 40% of casual and sex worker contacts) in Kisumu to fit recent falls in HIV prevalence (pc *C.Cohen*, 20/4/2007). Per-contact cofactor effects of other STIs on HIV susceptibility and infectivity were assumed to be 25 for chancroid, 7.5 for primary syphilis, and 3 for gonorrhoea and chlamydia infection. Lack-of-male-circumcision was assumed to double the per-contact probability of male acquisition of HIV, syphilis and chancroid [45,46]. In the model, in a sexual encounter in which there was more than one cofactor present, only the highest of the relevant cofactor effects was assumed to act on the probability of HIV transmission in that sexual act, because it is unclear how cofactors combine to increase transmission [47].

In this paper we present results for two cities. Cotonou, Benin represents an HIV epidemic highly concentrated among female sex workers (FSWs) and their clients, while Kisumu, Kenya represents a more generalised HIV epidemic typical of many countries in Eastern and Southern Africa [27].

### 2.2 Simulated interventions

As the existing vaccines are unlikely to be efficacious in Africa, we explored the impact of a potential prophylactic vaccine. HSV2 vaccination was targeted at a proportion of HSV2-uninfected individuals who were simulated to benefit for a fixed duration of time. We simulated three primary scenarios in which the vaccine was assumed to (i) reduce susceptibility to HSV2 acquisition, (ii) reduce HSV2 reactivation (frequency and duration of clinical reactivation and HSV2 infectivity during and between clinical reactivations), or (iii) both.

In each of these three primary scenarios we assumed three ‘strengths’ of the effect of the vaccine. In the ‘weak, ‘moderate’ and ‘strong’ vaccine efficacy scenarios, the values of parameters listed above were reduced by either 30%, 75% or 90%, respectively. For example, in the ‘weak vaccine efficacy (iii)’ scenario, we assumed a 30% reduction in susceptibility to HSV2 acquisition, a 30% reduction in clinical reactivation duration, a 30% reduction in clinical reactivation frequency, and a 30% reduction in HSV2 infectivity during and between HSV2 clinical reactivations.

We assumed a linear increase in the prevalence of vaccination over 5 years from 0% to 50%, 0% to 70% or 0% to 90% of targeted HSV2-uninfected individuals. We simulated routine annual vaccination of 14-year olds (chosen as the oldest children reliably in-school), and an initial ‘catch-up’ campaign targeting 15–29-year olds. Both groups were subjected to the linear increase in vaccination prevalence. We simulated two other scenarios in which (a) the vaccine was only efficacious in females, and (b) the catch-up campaign was omitted.

We simulated separate scenarios in which the duration of the vaccine effect was assumed to be 5 years, 10 years or lifelong. We assumed all individuals would receive only one course of immunisation during their lifetime. The simulated intervention was ongoing from 1/1/2008. Results were based on means over 200–1000 simulations.

### 2.3 Outcome

We present the percentage reduction in annual HSV2 and HIV incidence in adults aged 15–49-year old after 10 and 20 years, calculated by comparing intervention and baseline scenarios, and the number of vaccinations required to avert one HIV infection over 10 and 20 years.

### 2.4 Sensitivity analysis

We assessed the robustness of our findings to key baseline and intervention model parameter values known to affect impact. Full details are shown in the supporting information, online.

## 3 Results

### 3.1 Baseline scenario

**Fig. 1:**
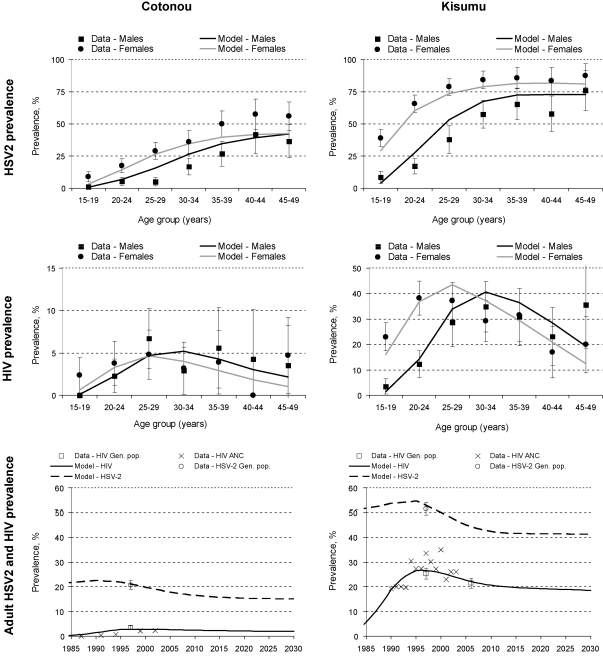
Observed (95% CI) and simulated prevalence of HIV and HSV2 by age and gender in 1997 and over time in Cotonou and Kisumu (adults aged 15–49 years). M, male; F, female. Note difference in *y*-axis scale used on HIV prevalence graphs. Gen. pop. = general population. ANC = ante-natal clinic.

Fig. 1 shows that a good fit of the model simulations to data for demography, sexual behaviour and epidemiology of the two populations was achieved, as published [23,27]. The simulated PAF for HSV2 among 15–49-year olds over 2008–2013 was 45% in Cotonou and 35% in Kisumu. Full details of the baseline scenario are shown in the supporting information, online.

### 3.2 Interventions

**Fig. 2:**
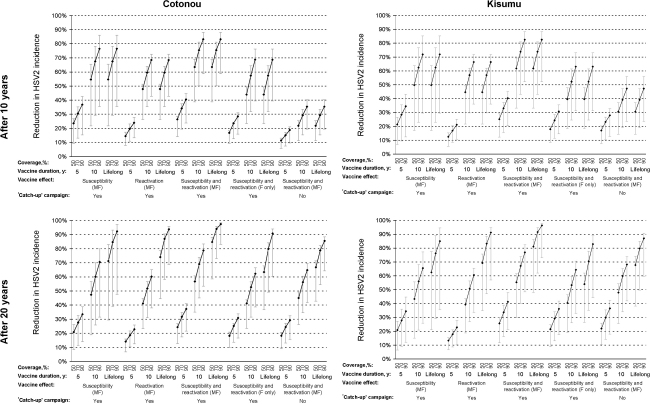
Impact of HSV2 prophylactic vaccines on HSV2 incidence after 10 and 20 years, by city (adults aged 15–49 years). For each combination of vaccine coverage (50%, 70%, 90%), vaccine duration of effect (5 years, 10 years, lifelong), vaccine effect (susceptibility or reactivation or both), and whether a ‘catch-up’ campaign of 15–29-year olds was simulated in addition to routine annual vaccination of 14-year olds, three scenarios are shown corresponding to the ‘weak’ (30%, ⊥), ‘moderate’ (75%, ♦) and ‘strong’ (90%, ⊤) vaccine efficacy. See Section 2 for full details. M, male; F, female.

**Fig. 3:**
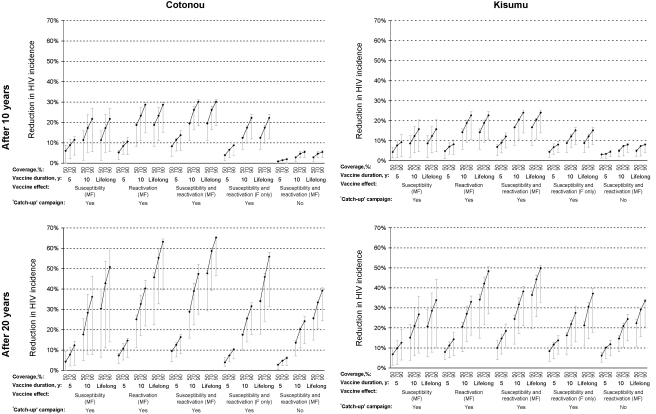
Impact of HSV2 prophylactic vaccines on HIV incidence after 10 and 20 years, by city (adults aged 15–49 years). For each combination of vaccine coverage (50%, 70%, 90%), vaccine duration of effect (5 years, 10 years, lifelong), vaccine effect (susceptibility or reactivation or both), and whether a ‘catch-up’ campaign of 15–29-year olds was simulated in addition to routine annual vaccination of 14-year olds, three scenarios are shown corresponding to the ‘weak’ (30%, ⊥), ‘moderate’ (75%, ♦) and ‘strong’ (90%, ⊤) vaccine efficacy. See Section 2 for full details. M, male; F, female.

The effects of the interventions in terms of percentage reduction in HSV2 and HIV incidence after 10 and 20 years in the two cities are shown in Figs. 2 and 3. The point estimates (black-markers) result from simulations assuming ‘moderate’ (75%) vaccine efficacy options for all parameters, and the plausible bound (grey-lines) from assuming ‘weak’ (30%) or ‘strong’ (90%) values. For clarity, it was assumed that there are no other changes in interventions, and no antiretroviral therapy, over the period.

Prophylactic vaccination that only reduced HSV2 susceptibility tended to have a slightly larger impact on HSV2 incidence than vaccination that only reduced HSV2 reactivation (Fig. 2). Vaccination that reduced both HSV2 susceptibility and reactivation had a slightly larger effect on HSV2 incidence, leading to reductions in HSV2 incidence of 12–98% across both cities after 20 years.

Conversely, a vaccination that only reduced susceptibility to HSV2 infection was predicted to have a slightly smaller effect on HIV incidence than a vaccination that only reduced HSV2 reactivation (Fig. 3). Again vaccination that reduced both HSV2 susceptibility and reactivation had a slightly larger effect on HIV incidence, leading to reductions in HIV incidence of 4–66% across both cities after 20 years.

As expected, the impact on HIV incidence increased with vaccination efficacy, duration of effect, and coverage (Fig. 3). A vaccination campaign that achieved 70% coverage over 5 years using a moderately effective vaccine with 10-year duration of effect on susceptibility and reactivation in both sexes would reduce HIV incidence by around 30–40% across both cities after 20 years.

Vaccination that was only efficacious in women would have around two-third of the impact on HIV incidence of vaccination that was efficacious in both genders (Fig. 3). Omitting an initial ‘catch-up’ campaign covering 15–29-year olds reduced impact. However, increasing the age range to include all HSV2-unifected individuals aged 30+ years had little additional effect on HIV (not shown). The impact of vaccination on incidence over 10 or 20 years (Figure S1/S2) was smaller than the corresponding impact on incidence after 10 and 20 years (Figure 1/2) because of the increasing contribution of indirect effects over time.

**Fig. 4:**
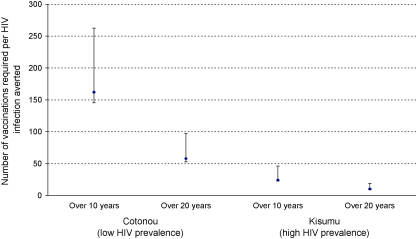
Number of vaccinations per HIV infection averted over 10 and 20 years, by city (all ages). The modelled scenario assumes an increase from 0% to 70% vaccine coverage over 5 years in 14–29-year olds using a vaccine with 10-year duration of effect on susceptibility and reactivation in males and females. Three scenarios are shown corresponding to the ‘weak’ (30%, ⊤), ‘moderate’ (75%, ♦) and ‘strong’ (90%, ⊥) vaccine efficacy. See Section 2 for full details.

The number of vaccinations required to avert one HIV infection was much lower in Kisumu than Cotonou, because of the higher HIV prevalence in Kisumu, but it fell over time in both sites because of ongoing indirect effects (i.e., preventing one infection now prevents multiple infections in the future) (Fig. 4). Over 20 years, a vaccination campaign that achieved 70% coverage over 5 years using a moderately effective vaccine with 10-year duration on susceptibility and reactivation in both sexes would avert one HIV infection for every 10 vaccinations in Kisumu and every 58 vaccinations in Cotonou, respectively. Over 20 years, in most scenarios fewer than 100 vaccinations were required to avert one HIV infection (not shown).

The effect of the interventions on prevalence after 20 years was smaller for HSV2 than HIV despite the larger impact of the interventions on HSV2 incidence, because HIV-infected individuals are removed more quickly from the population by AIDS mortality. Vaccination that reduced both HSV2 susceptibility and reactivation reduced HSV2 and HIV prevalence by 3–35% and 5–53%, respectively, across both cities after 20 years.

### 3.3 Sensitivity analysis

For HSV2 incidence, the sensitivity analysis of the effect of changing each intervention parameter value individually shows that the effect on HSV2 susceptibility had the largest effect. For HIV incidence, the effects of the vaccine on HSV2 susceptibility, clinical reactivation duration and clinical reactivation frequency were approximately as important as each other (Figure S3), while together, the three reactivation effects had a larger impact on HIV incidence than the susceptibility effect alone (Fig. 3).

Figure S4 shows the sensitivity of the results to the baseline parameter value assumptions made about cofactor effects in different stages of HSV2 infection. As expected, these changes had no impact on HSV2 incidence.

Assuming a between-clinical reactivation cofactor effect, while keeping the PAF for HSV2 constant by reducing the cofactor effects during clinical reactivation, reduced the impact of vaccination on HIV incidence in both cities. This is because the vaccine was assumed not to reduce between-clinical reactivation cofactor effects, so if this contributed to the total cofactor effect, the potential benefit of simulated vaccination was reduced.

Changing HSV2 clinical reactivation cofactors had larger impacts on the effects of vaccination on HIV incidence because they changed the PAF of HIV incidence associated with HSV2 (Figure S4). However, doubling or halving HSV cofactor effects does not provide as good a fit to the observed relative risks for the association of HSV2 and HIV [43].

## 4 Discussion

This study shows that HSV2 vaccination could theoretically reduce HIV incidence sufficiently for a substantial public health impact. While the largest impact on HIV incidence would be achieved if vaccination reduced both HSV2 susceptibility and reactivation, our results suggest that most of this impact would be achieved by a vaccine that only reduced susceptibility or reactivation. A larger relative impact was seen in Cotonou, the lower HIV prevalence city, because of the higher PAF for HSV2, although more vaccinations would be required to avert one infection in this lower HIV prevalence population.

In addition to the direct benefit of reducing HSV2-related morbidity, our results suggest that high efficacy HSV2 vaccines could have a substantial impact on population-level HIV incidence, and lower efficacy vaccines could still have a useful impact on HIV if given at high coverage. Vaccines inducing lifelong protection would be easier to implement because vaccination could be integrated into existing childhood vaccination schedules. If vaccines had shorter durations of effect they would need to be targeted at age groups just before their highest risk of HSV2 infection, such as women under 15 years and slightly older men.

The impact of HSV2 vaccination on HIV incidence and prevalence in the model is directly dependent upon the assumed strength of the relationship between HSV2 and HIV. The simulated relative risks and PAFs were in line with empirical data, but the failure of the two recent randomised-controlled trials of herpes suppressive therapy to show an impact on HIV acquisition [15,16] has cast doubt on this relationship [48]. If we have over- or under-estimated the PAF of HIV incidence associated with HSV2, then we may have over- or under-estimated the potential impact of vaccination on HIV incidence.

As our sensitivity analysis showed, the impact of vaccination was somewhat dependent on assumptions about the relative importance of clinical and sub-clinical reactivations for HIV transmission (Figure S4). However, vaccination might also reduce any sub-clinical cofactor effect so the sensitivity of our results to this uncertainty may have been overestimated. If sub-clinical reactivations were more important than we assumed and their frequency wanes more slowly than we assumed for clinical reactivations, then vaccine impact may take longer to appear, because prevalent HSV2 cases would continue to increase HIV transmission for longer after HSV2 infection. The slightly larger predicted impact of reduced HSV2 reactivation compared to HSV2 susceptibility should be treated with caution because it depends on the assumed effects of the potential vaccine. Understanding of HSV2 natural history continues to improve from empirical work and should be incorporated into future modelling studies.

It is important to note that the impact of a prophylactic vaccination campaign on HIV incidence over a period of time will always be smaller than the simulated PAF for HSV2 over the same time period, because prophylactic vaccination will not have a direct effect on the large proportion of individuals that are already HSV2-infected. For this a therapeutic vaccination or HSV therapy is required. Results of a trial of the impact of HSV2 therapy on HIV transmission will be available in 2009.

Specific assumptions about the action and mechanism of the interventions on HSV2 were informed by the literature wherever possible, but as this intervention was hypothetical, a wide range of values was explored. Even with these ranges, it is possible that our assumptions may have been overly optimistic in terms of some parameters, such as being able to vaccinate at least 50% of 14–29-year olds over 5 years, and being able to achieve a vaccine ‘strength’ at least as large as a 30% reduction in susceptibility, clinical reactivation duration and frequency, and HSV2 infectivity.

The most optimistic estimates suggests an HSV2 vaccine will not be available for at least 10 years (pc *A.Wald*, 8/4/2008). Most African countries should be providing affordable antiretroviral treatment (ART) within this period. ART reduces HSV2 reactivation in individuals with ART-induced reversal of immunosuppression and this is likely to reduce the impact of a potential HSV2 vaccine. However, even at high coverage, ART may not reduce HIV incidence in Africa [49] and therefore a HSV2 vaccine could still be a useful HIV prevention intervention.

We have presented the reductions in HIV incidence and prevalence that could be achieved through the introduction of a hypothetical HSV2 vaccination in certain scenarios. Should a vaccine become available, the real-world impact of scaled-up HSV2 interventions would be tempered by logistical constraints, but HSV2 vaccination has considerable potential as an HIV preventive intervention. Intensified efforts are needed to develop an effective vaccine against HSV2.
