# Supplementary material for: Population-level effect of potential HSV2 prophylactic vaccines on HIV incidence in sub-Saharan Africa
Source: Vaccine. 2009 Feb 5;27(6):940–6. doi: 10.1016/j.vaccine.2008.11.074 (PMC2686080; doi:10.1016/j.vaccine.2008.11.074)
Supplement: Supplementary file 1 [file mmc1.doc]

**ONLINE ONLY MATERIAL**

**Methods - Sensitivity analysis**

We assessed the robustness of our findings to key baseline model parameter values known to affect impact. We explored two scenarios in which we assumed a per-contact HIV cofactor effect between HSV2 clinical reactivations in both the ‘early’ and ‘middle’ stages of 2.0 or 3.0(cofactor=1.0 in baseline scenario). In these scenarios, to avoid over-estimating the total impact of HSV2 on HIV, the PAF for HSV2 was refitted to the mean of the simulated PAFs in Cotonou and Kisumu over 2008-2012 in the baseline scenarios(Cotonou=45%, Kisumu=35%,mean=40%), by reducing the magnitude of the cofactor effects assumed for primary genital herpes and clinical reactivations. We also simulated scenarios in which the assumed per-contact HIV cofactor effects for primary genital herpes and clinical reactivations were doubled or halved. In these scenarios we refitted the HIV prevalence in 1997 by varying the HIV transmission probabilities. In all scenarios the biological parameter values were kept constant across the two cites. See figure footnotes for fitted parameter values in alternative scenarios. We present this sensitivity analysis for both cities for the scenario in which a moderate efficacy vaccine produced lifelong effects on susceptibility and reactivation in both sexes with 90% coverage. This scenario was chosen because it resulted in a large impact in which the sensitivity of the results could be clearly shown.

To explore the sensitivity of our results to our assumed intervention parameter values, in the ‘weak’(30%) and ‘strong’(90%) vaccine efficacy scenarios separately, we changed the value of each vaccine efficacy parameter in turn to its ‘moderate’ value(75%). This method was selected because it highlighted the sensitivity of our results more clearly than if we varied only one intervention parameter in turn from its ‘moderate’ value.

**Results - Baseline scenario**

A good fit of the model simulations to data for demography, sexual behaviour and epidemiology of the two populations was achieved, as published[1, 2]. The fit of simulated HSV2 prevalence to data from the two cities can be seen in Figure 1 in the main text. Prevalence of HSV2 in younger age groups was fitted preferentially, because younger individuals account for a higher proportion of new HIV and HSV2 infections. Therefore HSV2 prevalence was underestimated in older females in Cotonou. In both sites, prevalence of HSV2 in younger males was allowed to be overestimated in favour of fitting the prevalence of HSV2 in younger females, because higher-risk males may not have been completely captured in the original surveys[3]. The fit of HSV2 prevalence reflects the general age trends in HSV2 prevalence and the important differences between the two sites, such as the difference in HSV2 prevalence in females aged 15–19 years in Cotonou(9%) and Kisumu(39%)[4]. Due to the sensitivity of simulated HIV prevalence and short-duration STI prevalence to the alteration of sexual behaviour parameters, these parameters could not be further altered to improve the fit for HSV2 without worsening the fit for HIV and the other STIs.

The model provided a reasonable fit to HIV prevalence by age and sex and over time in both sites (Figure 1 in the main text). The model replicated the observed patterns, including the much higher HIV prevalence among young females compared with young males. In Cotonou, HIV prevalence was very similar among males and females, as observed. For Kisumu the HIV prevalence peaked at a younger age in females than in males. HIV and HSV2 prevalences fell over time because of the simulated increase in condom use.

**Refs**

[1] Orroth KK, Freeman E, Bakker R, Buve A, Glynn J, Boily M-C, et al. Understanding differences across the contrasting epidemics in East and West Africa: results from a simulation model of the Four Cities Study. STI. 2007;83:i5-i16.

[2] Freeman E, Orroth K, White RG, Glynn JR, Bakker R, Boily M-C, et al. The proportion of new HIV infections attributable to HSV-2 increases over time: simulations of the changing role of sexually transmitted infections in sub-Saharan African HIV epidemics. STI. 2007;83:i17-i24.

[3] Buve A, Lagarde E, Carael M, Rutenberg N, Ferry B, Glynn JR, et al. Interpreting sexual behaviour data: validity issues in the multicentre study on factors determining the differential spread of HIV in four African cities. AIDS. 2001 Aug;15 Suppl 4:S117-26.

[4] Weiss H, Buvé A, Robinson N, et al. The epidemiology of HSV-2 infection and its association with HIV infection in four urban African populations. AIDS. 2001;15(suppl 4): S97-S108.

**Figure S1**

**Impact of HSV2 prophylactic vaccines on HSV2 incidence over 10 and 20 years, by city (adults aged 15-49 years).**  For each combination of vaccine coverage (50%, 70%, 90%), vaccine duration of effect (5 years, 10 years, lifelong), vaccine effect (susceptibility or reactivation or both), and whether a ‘catch-up’ campaign of 15-29 year olds was simulated in addition to routine annual vaccination of 14 year olds, three scenarios are shown corresponding to the ‘weak’ (30% , ┴ ), ‘moderate’ (75% , ♦ ) and ‘strong’ (90% , ┬ ) vaccine efficacy. See methods for full details. M, male; F, female.

|  | **Cotonou** | **Kisumu** |
| --- | --- | --- |
| Over 10 years | 5 10 Lifelong 5 10 Lifelong 5 10 Lifelong 5 10 Lifelong 5 10 Lifelong | 5 10 Lifelong 5 10 Lifelong 5 10 Lifelong 5 10 Lifelong 5 10 Lifelong |
| Over 20 years | 5 10 Lifelong 5 10 Lifelong 5 10 Lifelong 5 10 Lifelong 5 10 Lifelong | 5 10 Lifelong 5 10 Lifelong 5 10 Lifelong 5 10 Lifelong 5 10 Lifelong |

**Figure S2**

**Impact of HSV2 prophylactic vaccines on HIV incidence over 10 and 20 years, by city (adults aged 15-49 years).**  For each combination of vaccine coverage (50%, 70%, 90%), vaccine duration of effect (5 years, 10 years, lifelong), vaccine effect (susceptibility or reactivation or both), and whether a ‘catch-up’ campaign of 15-29 year olds was simulated in addition to routine annual vaccination of 14 year olds, three scenarios are shown corresponding to the ‘weak’ (30% , ┴ ), ‘moderate’ (75% , ♦ ) and ‘strong’ (90% , ┬ ) vaccine efficacy. See methods for full details. M, male; F, female.

|  | **Cotonou** | **Kisumu** |
| --- | --- | --- |
| Over 10 years | 5 10 Lifelong 5 10 Lifelong 5 10 Lifelong 5 10 Lifelong 5 10 Lifelong | 5 10 Lifelong 5 10 Lifelong 5 10 Lifelong 5 10 Lifelong 5 10 Lifelong |
| Over 20 years | 5 10 Lifelong 5 10 Lifelong 5 10 Lifelong 5 10 Lifelong 5 10 Lifelong | 5 10 Lifelong 5 10 Lifelong 5 10 Lifelong 5 10 Lifelong 5 10 Lifelong |

**Figure S3**

**Sensitivity analysis of the impact of prophylactic vaccines to intervention parameter values.** The default scenarios here show the impacts after 20 years of a vaccine producing lifelong effects on susceptibility and reactivation in both sexes with 90% population coverage. In the ‘weak’(30%) and ‘strong’(90%) vaccine efficacy scenarios separately, the value of each vaccine efficacy parameter has been changed in turn to its ‘moderate’ value(75%). Note difference in y-axis scale.

|  | Cotonou | Kisumu |
| --- | --- | --- |
| Reduction in HSV2 incidence  after 20 years |  |  |
| Reduction in HIV incidence  after 20 years |  |  |

**Figure S4**

**Sensitivity analysis of the impact of prophylactic vaccines to baseline parameter values.** The default scenarios here show the impacts after 20 years of a ‘moderate’ efficacy vaccine producing lifelong effects on susceptibility and reactivation in both sexes with 90% population coverage. Note difference in y-axis scale. As described in the methods, after increasing the between-clinical reactivation cofactor effects the models were refitted by decreasing the clinical reactivation cofactors effects. When the between-clinical reactivation cofactor was set to 2, the fitted cofactor effect magnitudes were 17.9 for primary genital herpes and 7.1 for recurrent clinical reactivations; and with a between-clinical reactivation cofactor of 3 the clinical cofactors were 7.0 for primary genital herpes and 4.3 for recurrent clinical reactivations. Similarly, when assessing the effect of doubling or halving the clinical-reactivation cofactor effect, the model was refitted by changing the per-contact HIV transmission probabilities. The resulting mean probabilities were 0.39% from males to females and 0.20% from females to males when clinical cofactors were doubled, and 0.47% from males to females and 0.23% from females to males when clinical cofactors were halved.

|  | Cotonou | Kisumu |
| --- | --- | --- |
| Reduction in HSV2 incidence  after 20 years |  |  |
| Reduction in HIV incidence  after 20 years |  |  |
